# Unilateral linear syringoma on the right chest and arm^☆^

**DOI:** 10.1016/j.abd.2021.09.017

**Published:** 2022-11-01

**Authors:** Danyi Huang, Yanqing Chen, Jianjian Li, Han Ma

**Affiliations:** Department of Dermatology, The Fifth Affiliated Hospital of Sun Yat-sen University, Zhuhai, Guangdong, China

Dear Editor,

A 25-year-old man presented to the dermatology department with an 8-year history of yellowish-brown skin lesions on his right chest, axilla, and arm. The lesions first appeared on the right chest and increased in number and size gradually, showing a linear distribution, with no pain or itching. He was otherwise healthy with no other special medical history. Physical examination revealed a yellowish-brown firm, smooth papules and plaques on his right chest, axilla, and arm, ranging from 1 to 10 mm in diameter (Figs. 1–4 ). The distribution of the lesions followed Blaschko's lines. Histopathology showed multiple small tubules, cysts, and nests forming by epithelial proliferation, with some of them morphologically like a comma or a tadpole, in the superficial dermis (Fig. 5). A diagnosis of sporadic linear syringoma was made. As it is a benign disease with no malignant potential, the patient has been followed up regularly without any treatment.

**Fig. 1:**
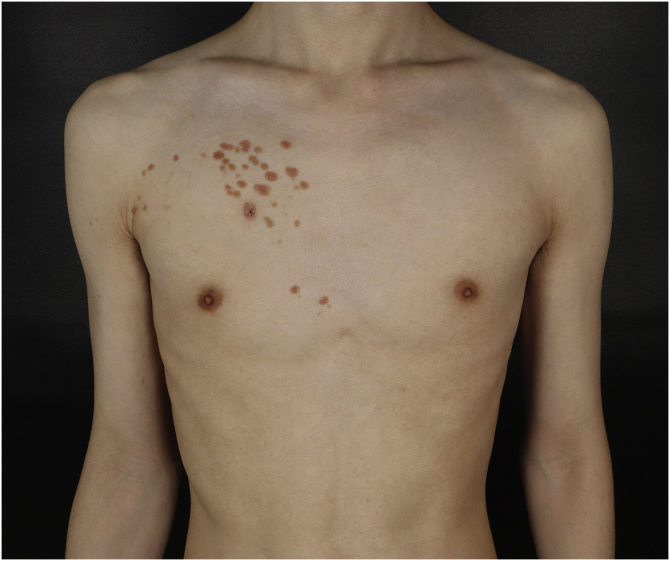
Physical examination revealed yellowish-brown firm, smooth papules, and plaques on his right chest. Distribution of the lesions followed Blaschko's lines.

**Fig. 2:**
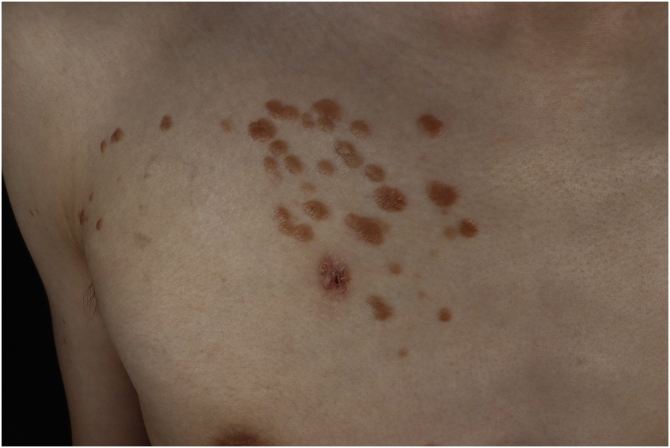
Closer look on the right chest. There is a wound after skin biopsy at the bottom.

**Fig. 3:**
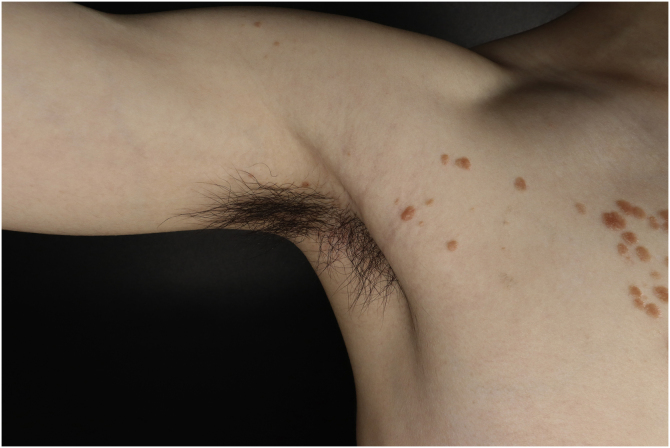
Lesions on the axilla.

**Fig. 4:**
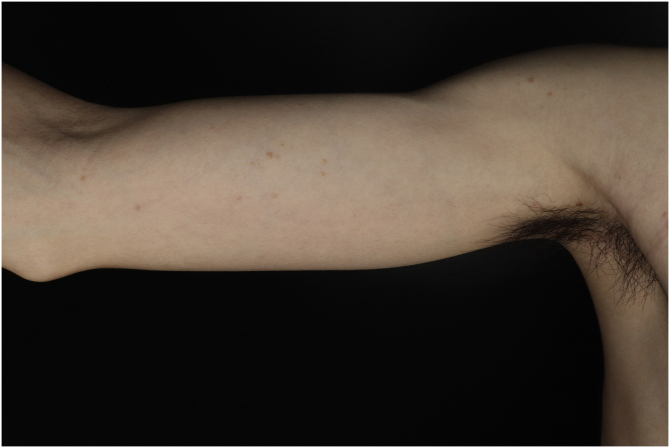
Lesions on the right upper limb.

**Fig. 5:**
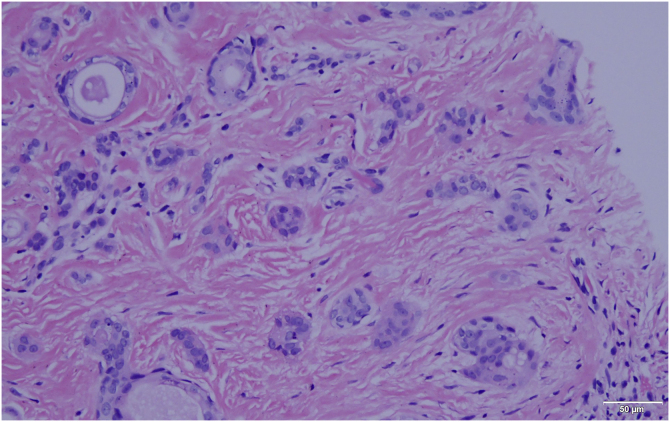
Histopathology showed multiple small tubules, cysts, and nest forming by epithelial proliferation, with some of them morphologically like a comma or a tadpole, in the superficial dermis (Hematoxilin & eosin, X40).

## Discussion

Syringoma is a benign neoplasm that derives from eccrine sweat gland ducts. The typical clinical manifestation is single or multiple skin-color or yellowish papules on the lower eyelid, 1 to 3 mm in size. Friedman and Butler classified syringoma into 4 clinical variants: generalized forms, localized forms, familial forms and variants associated with Down’s syndrome.^1^ Differential diagnosis of syringoma based on a variety of clinical manifestations includes milia, xanthoma, hidrocystoma, trichoepithelioma, and flat warts, especially for the eyelids; other considerations include cutaneous mastocytosis, fibrofolliculomas, vellus hair cysts, angiofibroma, and fibroelastic papulosis, lichen planus, steatocystoma multiplex, eruptive vellus hair cysts, disseminated granuloma annulare, secondary syphilis and so on.^2^, ^3^ Diagnosis can be confirmed by typical histopathologic manifestations.

Though syringoma is a common ailment, the pathogenesis remains unclear, some investigators have suggested that syringomas may be caused by hormonal factors.^4^ The linear arrangement along Blascko’s lines is unusual and indicated the genetic association in pathogenesis.^5^ However, we could not find any congenital abnormalities in the present case.

As we all know, syringoma rarely shows the unilateral or linear distribution. There have been only 4 reports of unilateral linear syringoma, all involving the upper trunk and limb.^6^, ^7^, ^8^, ^9^ We learned from previously reported cases that linear syringoma usually presents clinically quite similar to ordinary syringoma. Nevertheless, in the present case, the size of most skin lesions was larger than the usual size and the color was different, which reminds dermatologists of the necessity to consider syringoma in the differential diagnosis of unilateral linear papular or plaque skin lesions.

## Financial support

This work is supported by Medical Science and Technology Research Projects in Guangdong Province (A2021365) and Science and Technology Plan Projects of Zhuhai (ZH2202200003HJL).

## Authors' contributions

Danyi Huang took effective participation in research orientation and completed the preparation and writing of the manuscript. Yanqing Chen was in charge of the data collection, analysis and interpretation. Jianjian Li helped with data collection. Han Ma formed the study conception and planning and approved the final version of the manuscript.

## Conflicts of interest

None declared.
